# Supplementary figures and images for: Synthesis of 1-Amino-3-oxo-2,7-naphthyridines via Smiles Rearrangement: A New Approach in the Field of Chemistry of Heterocyclic Compounds
Source: Int J Mol Sci. 2022 May 25;23(11):5904. doi: 10.3390/ijms23115904 (PMC9179986; doi:10.3390/ijms23115904)

ha-979-3

Feb 22 2021 ANUSH\_TEMA ha-979-3

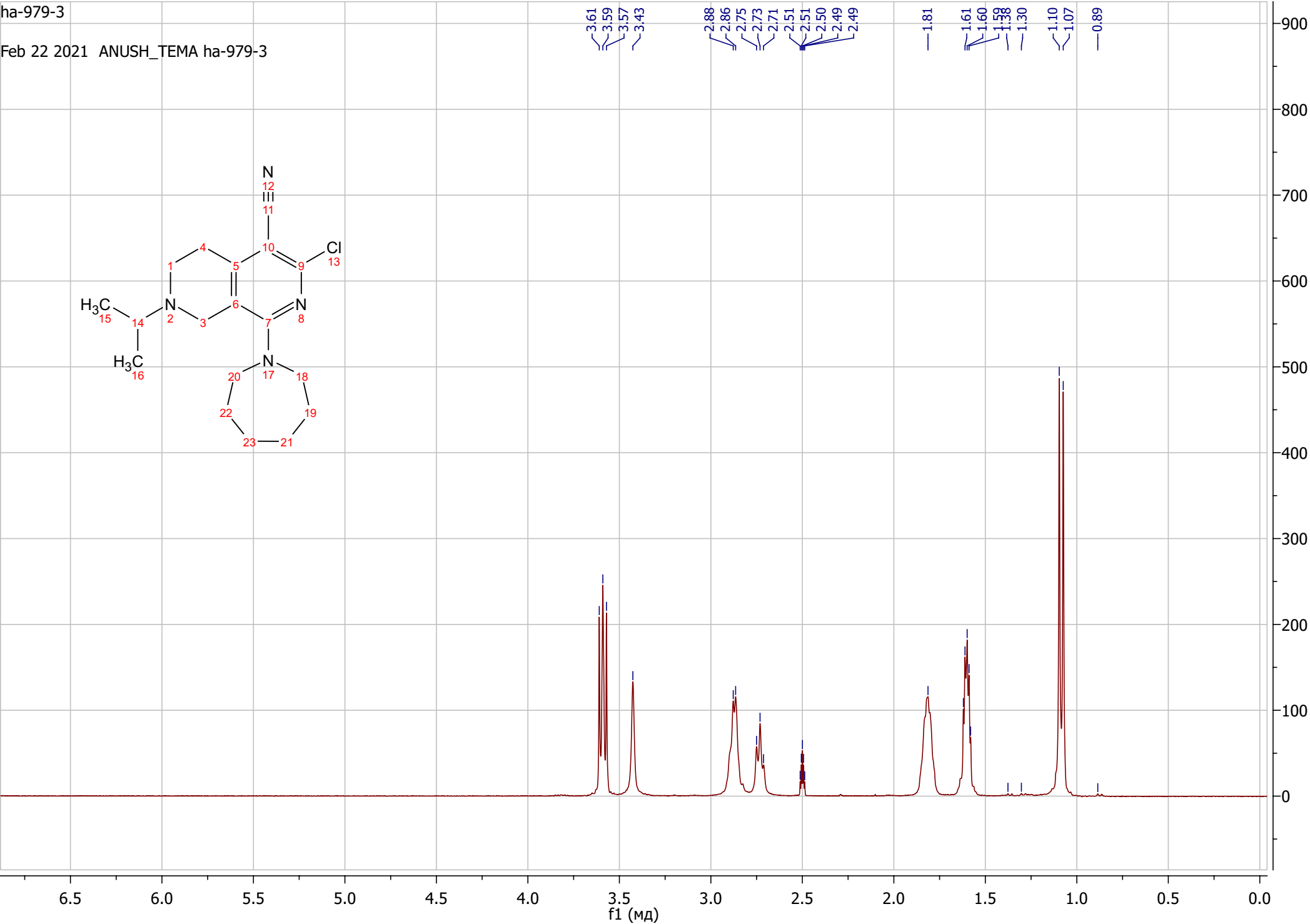

Supplement: Supplementary file 1 [file ijms-23-05904-s001.zip › ijms-1703034-supplementary.pdf]
